# New recurrent BRCA1/2 mutations in Polish patients with familial breast/ovarian cancer detected by next generation sequencing

**DOI:** 10.1186/s12920-015-0092-2

**Published:** 2015-05-07

**Authors:** Anna Kluska, Aneta Balabas, Agnieszka Paziewska, Maria Kulecka, Dorota Nowakowska, Michal Mikula, Jerzy Ostrowski

**Affiliations:** Department of Genetics, Maria Sklodowska-Curie Memorial Cancer Center and Institute of Oncology, 02-781 Warsaw, Poland; Department of Gastroenterology, Hepatology and Clinical Oncology, Medical Center for Postgraduate Education, 01-813 Warsaw, Poland

## Abstract

**Background:**

Targeted PCR-based genetic testing for *BRCA1* and *BRCA2* can be performed at a lower cost than full gene testing; however, it may overlook mutations responsible for familial breast and/or ovarian cancers. In the present study, we report the utility of next generation sequencing (NGS) to identify new pathogenic variants of *BRCA1/2*.

**Methods:**

*BRCA1* and *BRCA2* exons were amplified using the Ion AmpliSeq BRCA1/2 Panel and sequenced on the Ion Torrent PGM sequencer in 512 women with familial and/or only early onset breast and/or ovarian cancers who were negative for selected *BRCA1/2* mutations.

**Results:**

146 single nucleotide variants (SNVs) and 32 indels were identified. Of them, 14 SNVs and 17 indels were considered as pathogenic or likely pathogenic. One and 18 pathogenic mutations had been detected previously in the Polish and other populations, respectively, and 12 deleterious mutations were previously unknown. Eight mutations were recurrent; Q563X (*BRCA1*), N3124I (*BRCA2*) and c.4516delG (*BRCA1*) were found in eight, six and four patients, respectively, and two other mutations (c.9118-2A > G and c.7249delCA in *BRCA2*) were detected in three patients each. Altogether, *BRCA1/2* pathogenic mutations were identified in 52 out of 512 (10%) patients.

**Conclusions:**

NGS substantially improved the detection rates of a wide spectrum of mutations in Polish patients with familial breast and/or ovarian cancer. Although targeted screening for specific *BRCA1* mutations can be offered to all Polish breast or ovarian cancer patients, NGS-based testing is justified in patients with breast or ovarian cancer likely related to *BRCA1/2* who test negative for the selected *BRCA1/2* pathogenic mutations.

**Electronic supplementary material:**

The online version of this article (doi:10.1186/s12920-015-0092-2) contains supplementary material, which is available to authorized users.

## Background

Approximately 5–10% of breast cancers and 10% of ovarian cancers have a hereditary component [1], which in most cases is associated with clinically significant mutations in the *BRCA1* and *BRCA2* genes. According to the Breast Cancer Information Core (BIC) [2], more than 1800 and 2000 distinct variants of the *BRCA1* and *BRCA2* genes have been described, respectively. The incidence and spectrum of mutations vary among populations, and the carrier frequency in general populations ranges between 1/40 and 1/800 [3]. Some populations demonstrate a wide spectrum of mutations, while others are characterized by high prevalence of a small number of founder mutations [4]. In Ashkenazi Jews, three founder mutations in *BRCA1* and *BRCA2* comprise 80% to 95% of mutations, respectively [5,6]. In Poland, the frequency of *BRCA1* mutations is estimated to be between 1/240 and 1/360, and two founder mutations, namely, 5382insC and T300G (C61G), account for 70–90% of *BRCA1* mutations [3]. None of them is unique to Poland.

Breast cancer is the most common cancer among Polish women, and is a leading cause of cancer-related morbidity and mortality [7]. The only factor that significantly decreases cancer-related mortality is early diagnosis, and in special cases, prophylactic surgery. Since cancers detected at an early stage of development are asymptomatic or low-symptomatic disorders, early diagnosis of cancer is usually accidental or the result of screening programs. Current cancer screening guidelines for the general population are based on the age of the individual, and patients at risk of developing cancer are identified on the basis of a positive family history of cancer. Families with two first- or second-degree relatives with breast and/or ovarian cancers, with at least one of the cases diagnosed before the age of 50 years, are considered to be at moderate risk, while those with at least three relatives with breast and/or ovarian cancers are considered to be at high risk [7].

The mutation status may determine the medical management of patients, including annual screening and prophylactic surgery, and genetic testing for *BRCA1* and *BRCA2* is routinely performed in women with hereditary breast and ovarian cancer risk. More than 100,000 individuals in the United States undergo BRCA testing annually [8]. However, since BRCA testing is a costly procedure (the current charge for full gene sequencing is several hundred to thousands of dollars), most genetic counseling programs recommend less expensive targeted screening for specific *BRCA1* and *BRCA2* mutations rather than full gene testing [8].

Next generation sequencing (NGS) offers a powerful, faster, and cheaper alternative for targeted sequencing aimed at identifying non-synonymous, splicing, and stop codon variants exhibiting deleterious consequences in the coding genes [9]. As a consequence, several NGS-based workflows for clinical *BRCA1* and *BRCA2* mutation testing were recently proposed [10-16].

In a previous study from our group, we used multiplexed PCR-based genotyping technology and showed that the frequency of selected *BRCA1* mutations was moderate in familial and high in nonfamilial breast cancers. In addition, a strong founder effect was confirmed for *BRCA1* mutations but not for *BRCA2* mutations in the Polish population [7]. In the present study, we show that the use of the Ion Torrent PGM sequencer to sequence the coding exons of *BRCA1* and *BRCA2* genes substantially improves the detection rates of a wide spectrum of mutations in Polish patients with familial and/or only early onset of breast and/or ovarian cancers.

## Methods

### Patients

Patients whose blood samples were collected between 2003 and 2010 were selected from the registry of the Genetic Counseling Unit, Warsaw Cancer Center-Institute of Oncology. The personal and familial cancer histories were acquired by in-depth interviews. Healthy individuals with no known personal or familial history of malignancy, normal results of screening colonoscopy, and normal mammography or PSA levels were recruited primarily from the National Colorectal Cancer Screening Program. All patients and control subjects were Polish Caucasians recruited from the Masovian voivodeship population. Informed consent for hereditary cancer genetic testing was obtained from all of the patients. The permission for genetic testing was granted by the Ethical Committee at Maria Sklodowska-Curie Memorial Cancer Center and Institute of Oncology on 9 May 2002 (No. 28/2002) and further extended to include testing with NGS on 11 June 2013 (No. 28/2002/1/2013). The study did not require ethical approval.

### BRCA1/2 sequencing

Genomic DNA was extracted from whole blood as described previously [7]. DNA concentration was determined on a Qubit 2.0 Fluorometer using the Qubit dsDNA BR Assay Kit (Life Technologies). For library preparation, a set of reagents from the Ion AmpliSeq™ Library Kit 2.0 (Life Technologies) and Ion AmpliSeq BRCA1 and BRCA2 Panel, which comprises 167 primer pairs spanning 16.25 kb, were used to amplify the coding regions of the *BRCA1* and *BRCA2* genes. Amplification for each patient was performed using three separate primer pools containing 1.4 μl of 5× Ion AmpliSeq™ HiFi Master Mix, 3.5 μl of 2× Ion AmpliSeq Primer Pool, and 3 ng of DNA in a total reaction volume of 7 μl. Cycling conditions were as follows: 99°C for 2 min, followed by 19 cycles of 99°C for 15 sec and 60°C for 4 min, ending with a hold at 10°C. At this point, three separate reactions for a given patient were combined, and amplicons were partially digested at primer sequences with 2 μl of FuPa reagent under conditions provided by the manufacturer in The Ion AmpliSeq DNA and RNA Library Preparation protocol (Revision B.0). Subsequent steps of library preparation were performed according to the above protocol. Briefly, following digestion, samples were subjected to sequencing adapter ligation with either 16 or 32 indexing barcodes, and purified with the AMPure XP reagent (Beckman Coulter). The DNA library was eluted from AMPure XP beads with 52 μl of PCR reagent solution consisting of 50 μl of Platinum®PCR Super Mix HiFi and 2 μl of Library Amplification Primer Mix, and amplified under the following conditions: 98°C for 2 min, followed by five cycles of 98°C for 15 sec and 64°C for 1 min, ending with a hold at 10°C. Next, the amplified DNA library was subjected to a two-round purification process using AMPure XP beads with 0.5× and 1.2× bead-to-sample volume ratios, respectively. Library concentrations were determined with the Qubit dsDNA HS Assay Kit (Life Technologies), and the respective size distributions were determined with the High Sensitivity DNA Analysis Kit on Bioanalyzer 2100 (Agilent).

Library molarity was determined, and either 16 or 32 libraries were pooled in equimolar concentrations and used for automatic template preparation on Ion Chef using reagents from the Ion PGM IC 200 Kit and Ion 316 Chip Kit v2 BC. Sequencing was performed on the Ion Torrent PGM sequencer using 500-flow runs. Data from the PGM runs were processed on the Ion Torrent server using a platform-specific pipeline incorporated in Torrent Suite v4.2.1 (Life Technologies) to obtain sequence reads, trim adapter sequences, filter and remove poor signal reads, and assign the reads to a given barcode. The reads were mapped to the hg19 (*Homo sapiens*) reference genome and adjusted to the specific amplicon target regions deposited in the “BRCA1_2.20131001.designed” BED file available in Torrent Suite. The coverageAnalysis (v4.2.1.4) and variantCaller (v4.2.1.0) plug-ins with a set of default parameters optimized for the BRCA1/BRCA2 panel were obtained from www.Ampliseq.com, and were ran for each sequencing dataset.

### Post-sequencing variant analysis

Variants were classified either as single nucleotide variants (SNVs) or insertion/deletion (indels) variants using a python script. Variants were annotated using data from BIC for the *BRCA1* and *BRCA2* genes. The identified variants were matched to existing variants in BIC according to four parameters as follows: chromosome number (chromosome 13 and chromosome 17 for *BRCA2* and *BRCA1*, respectively); position of the variant in the human genome assembly hg19 (GRCh37), which was retrieved from the BIC database field HGVS; genomic and type of reference; and alternate base. The possibility of different alternate and reference base was taken into account as well as the possibility of variant on the reverse strand.

All the SNVs were analyzed using Variant Effect Predictor [17] (VEP) for the human genome assembly hg19 (GRCh37). The occurrence of variants was determined using an R script. Variants with unknown clinical significance in BIC and new changes were subjected to *in silico* analysis of their deleteriousness, and screened for evidence of pathogenicity in the literature.

### Selection of pathogenic variants

SNVs and indels were analyzed using Condel [18] and SIFT Indel [19], respectively. Missense variants were classified as pathogenic when reported in BIC as pathogenic, and/or predicted as pathogenic by a Condel score, and identified through a literature search [20-29]. Frameshift mutations were classified as pathogenic when reported as pathogenic by BIC and/or predicted as pathogenic by SIFT Indel.

### Genotyping

Pathogenic mutations detected by NGS were confirmed by Sanger sequencing. In addition, five selected variants, namely, Q356R, N3124I, Q563X, c.7249delCA, and c.9118-2G > A, were genotyped using Custom TaqMan SNP Genotyping Assays (Life Technologies, USA) and a 7900HT Real-Time PCR system (Life Technologies) as described previously [7].

## Results

### NGS analysis

The incidence of SNVs and indels within 167 amplicons covering the *BRCA1* and *BRCA2* exons was tested using the Ion Torrent PGM sequencer in 31 (a training set) and 512 women with breast cancer or ovarian cancer newly diagnosed before the age of 50 years who were positive and negative for *BRCA1/2* mutations, respectively, as determined by targeted genotyping. The genotyping comprised 11 mutations in *BRCA1*, namely c.66_67delAG, C61R, c.3700_3704del5, c.3756delGTCT, c.3777delT, c.4035delA, c.4041delAG, c.4065delTCAA, c.5263delC, R1738E and R1751X, and nine mutations in *BRCA2*, namely E394X, c.5239insT, c.5946delT, c.5964delAT, c.6447delTA, c.7910del5, c.8924delT, R3128X and c.9402delT [7,30]. Of the 512 women, 317 had familial breast and/or ovarian cancer, and 195 had only early onset cancer and/or contralateral breast and ovarian cancers; the median age of women with breast cancer at diagnosis was 43 years.

The average number of bases with ≥ Q17/≥Q20 across all 21 PGM sequencing runs was 343/324 Mbp, constituting 96/90% of total output. The average coverage across all samples was 507, whereas the mean mapping rate reached 94%.

The NGS-based procedure confirmed the pathogenic mutations in all 31 DNA samples of a training set (not shown). Among 512 DNA samples analyzed, we identified 146 SNVs (64 in *BRCA1* and 82 in *BRCA2*) and 32 indels (20 in *BRCA1* and 12 in *BRCA2*). Sixteen indels were found in coding regions (nine in *BRCA1* and seven in *BRCA2*). The results of the detailed analysis of SNVs and indels are summarized in Additional file 1.

The types and number of SNVs are summarized in Table 1. Of these, eight SNVs (five in *BRCA1* and three in *BRCA2*) were already reported in BIC as pathogenic. Three additional SNVs were reported as variants of uncertain significance (VUS); variants co-localized with these SNVs were reported as pathogenic in ClinVar. In addition, we identified three novel nonsense variants (Table 2). Of 41 missense variants that were either reported as VUS in BIC or had not been reported previously, 24 had at least one transcript in which the Condel score was reported as deleterious (Table 3).

**Table 1:** **Types and number of single nucleotide variants in the** ***BRCA1*** **and** ***BRCA2*** **genes**

**Type of variant**	**Number**	
	**BRCA1**	**BRCA2**
Intronic variant	21	23
Intronic variant near splice site	3	4
Missense variant	25	36
Nonsense variant	6	3
Synonymous variant	4	14
Variant in the splice region of exon	0	1
3′ or 5′ UTR variants	5	1

**Table 2:** **Pathogenic single nucleotide variants in BIC and ClinVar**

**Variant**	**Type**	**Evidence of pathogenicity**	**Count**
**BRCA1**			
Q563X *	Nonsense	BIC, ClinVar	8
W1782X	Nonsense	BIC, ClinVar	2
Y1563X	Nonsense	BIC, ClinVar	1
Q538X	Nonsense	BIC, ClinVar	1
E1415X	Nonsense		1
C1501X	Nonsense		1
R1699W	Missense	BIC, ClinVar	2
**BRCA2**			
Q92X	Nonsense	BIC, ClinVar	1
c.9118-2A > G	IVS	BIC, ClinVar	3
P3039P	Splice	BIC, ClinVar	1
Q3047X	Nonsense		1
N3124I	Missense	ClinVar, Condel	6
I2627F	Missense	ClinVar, Condel	1
c.8754 + 5G > T	IVS	ClinVar	2

* Patients with ovarian and breast cancer.

*IVS* intronic variant near splice site; *Splice* variant in the splice region of exon.

**Table 3:** **Pathogenic missense variants according to the Condel algorithm**

**Variant**	**Gene**	**rs**	**Count**	**Condel count**	**Condel max**	**Condel algorithms**	**Confirmed by other models**
Q356R	BRCA1	rs1799950	72	12	0.70898	4	no
S1040N	BRCA1	rs4986852	11	7	0.66416	3	no
K1690N	BRCA2	rs56087561	10	2	0.536316	2	no
N3124I	BRCA2	rs28897759	6	2	0.618793	4	yes
R1347G	BRCA1	rs28897689	6	7	0.540272	2	no
Q2858H	BRCA2	-	3	2	0.539229	3	novel
V2969M	BRCA2	rs59004709	2	2	0.644176	3	no
D1152N	BRCA1	rs80357175	2	7	0.596261	2	no
T1011R	BRCA2	rs80358548	1	2	0.531615	2	no
E2236K	BRCA2	rs41293503	1	2	0.591769	2	no
S2326C	BRCA2	-	1	2	0.599866	2	novel
I2627F	BRCA2	rs80359014	1	2	0.663608	4	yes
L2865F	BRCA2	-	1	2	0.593408	3	novel
V1791L	BRCA1	rs145758886	1	11	0.574356	3	no
Y1703C	BRCA1	-	1	14	0.622922	4	novel
A1669S	BRCA1	rs80357087	1	13	0.540083	3	no
M1628T	BRCA1	rs4986854	1	13	0.526967	1	no
E1346K	BRCA1	rs80357407	1	7	0.540139	1	no
R866H	BRCA1	rs80356911	1	7	0.676062	4	no
N723D	BRCA1	rs4986845	1	7	0.543268	1	no
N550H	BRCA1	rs56012641	1	9	0.681765	4	no
F486L	BRCA1	rs55906931	1	9	0.558913	1	no
Y179C	BRCA1	rs56187033	1	20	0.588385	4	no
G602R	BRCA1	-	1	14	0.660547	4	novel

Condel count: number of transcripts in which a given variant is deleterious; Condel max: maximal score from Condel; Condel algorithms: maximum number of algorithms (used in Condel score computation) in which a given variant is marked as deleterious.

Of the indels identified in the present study, five in *BRCA1* and two in *BRCA2* had already been reported in BIC as pathogenic. One additional indel was reported as unknown in BIC, although its co-located variants in dbSNP were reported in ClinVar as pathogenic (Table 4). We also found nine novel indels in coding regions (Table 4).

**Table 4:** **Pathogenic indels in BIC, ClinVar, or SIFT indel prediction**

**Variant**	**rs**	**Type**	**BIC**	**ClinVar**	**SIFT**	**Count**
	**BRCA1**					
c.4516delG	rs273900736	F	pathogenic		not-tested	4
c.5137delG	rs80357997	F	pathogenic	pathogenic	not-tested	1
c.1695insG	rs273897664	F	pathogenic		not-tested	1
c.340delTC	rs80357881	F	pathogenic		not-tested	1
c.5285insG	rs80357886	F	pathogenic		not-tested	1
c.4597delGA		F			pathogenic	1
c.5232del7ins12		F			pathogenic	1
c.403delA		F			pathogenic	1
c.403delAAGA		F			pathogenic	1
	**BRCA2**					
c.7249delCA		F			pathogenic	3
c.475 + 4delT	rs276174848	IVS	unknown	likely pathogenic	not-tested	1
c.696delT	rs80359630	F	pathogenic		not-tested	1
c.2808delACAA	rs80359352	F	pathogenic		not-tested	1
c.1318delCTTA		F			pathogenic	1
c.8840delA		F			pathogenic	1
c.8942delA		F			pathogenic	1
c.9375delC		F			pathogenic	1

*IVS* intronic variant near splice site, *F* frameshift variant.

Altogether, among 512 patients in whom the previous targeted genotyping did not reveal a pathogenic *BRCA1/2* mutation massive amplicon sequencing identified 52 patients with 31 deleterious mutations, of which sixteen were frameshift, eight were nonsense, three were missense, and four affected splicing. Of note, one patient carried two pathogenic mutations, namely Q563X and N3124I. All pathogenic variants were confirmed by Sanger direct sequencing. Five other variants were likely pathogenic, and 17 variants were VUS. All of them were missense mutations (Table 3).

### Genotyping for NGS-selected mutations

Using the TaqMan SNP Genotyping Assay, the incidence of five selected variants, one common polymorphism (Q356R) and four mutations (N3124I, Q563X, c.7249delCA, and c.9118-2G > A) were tested in additional group of 445 cancer patients diagnosed under the age of 50 and 1300 healthy individuals (856 women and 444 men with a median age of 59 years).

Although Q356R was described as pathogenic in ClinVar, its prevalence was similar among patients (15.1%) and healthy controls (16.9%). Therefore, the clinical significance of this polymorphic variant was not confirmed. By contrast, Q563X was detected in 5 (1.1%) out of 445 cancer patients, respectively, but only in one healthy individual. The three other mutations (N3124I, c.7249delCA, and c.9118-2G > A) were detected by TaqMan genotyping only in DNA samples in which they were identified by NGS.

## Discussion

The rare germline pathogenic *BRCA1* mutations increase the risk of breast and ovarian cancer to approximately 80% and 60%, respectively, whereas *BRCA2* mutations increase breast cancer risk to more than 80% by the age of 80 [10]. Because the risk of cancer can be predicted by a family history and/or by an early disease onset, the Genetic Counseling Unit at the Warsaw Cancer Center-Institute of Oncology offers risk counseling based on the occurrence of cancer in the pedigree. However, according to Polish guidelines and recommendations, the government sponsored surveillance program, which includes annual mammography and breast magnetic resonance imaging, pelvic ultrasonography, and cancer antigen 125 levels, is provided only to women with known pathogenic mutations. Therefore, in families with an increased cancer risk, genetic testing is critical for preventive healthcare, including specialized surveillance programs and prophylactic mastectomy and/or oophorectomy, which reduce the risk of breast/ovarian cancer by over 90% [31]. The detection of *BRCA1/2* pathogenic mutations facilitates the selection of patients and the delivery of healthcare and related services in women harboring their respective germline mutations.

Although *BRCA1* and *BRCA2* mutations [32-37] are present in more than 60% and 30% of families, respectively, our targeted mutation screening [7] detected 20.8% and 1.3% of patients carrying pathogenic *BRCA1* and *BRCA2* mutations, respectively. This suggests that a proportion of mutations responsible for familial breast and/or ovarian cancers were not identified. In the present study, we report the utility of NGS for the detection of new pathogenic variants of *BRCA1/2*.

**Figure 1:**
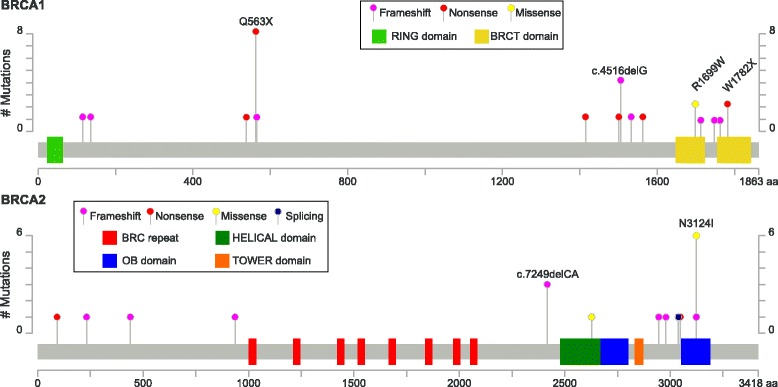
Mutations found in *BRCA1* and *BRCA2* genes. Visualization was performed with MutationMapper [55]. The Pfam protein domains and the positions of specific mutations are shown. The mutations found in two or more cases are labeled.

Among 512 DNA samples analyzed, we identified 146 SNVs and 32 indels, of which 14 SNVs and 17 indels were considered as pathogenic or likely pathogenic. One and 18 pathogenic mutations had been detected previously in the Polish and other populations, respectively, and 12 deleterious mutations had not been detected earlier. Eight mutations were recurrent; Q563X, N3124I and c.4516delG were found in eight, six and four patients, respectively, and two other mutations (c.9118-2A > G and c.7249delCA) were detected in three patients each (Figure 1). Overall, sequencing of *BRCA1/2* coding exons identified additional predicted cancer risk mutations in 52 out of 512 (10%) patients with familial and/or an early onset breast/ovarian cancer that were missed using our standard genotyping procedure. The incidence of new pathogenic mutations was higher in women with familial breast and/or ovarian cancer [26 mutations (17.9%) in 145 patients] than those representing only familial breast cancer [26 (7.1%) in 367 patients]. These mutations could be used for estimation of cancer risk in index patients and for cascade screening.

The pathogenicity of nonsense and frameshift mutations was relatively easily established; however, the functional consequences of several missense, intronic, and small in-frame insertion/deletions were not as evident, and reaching an adequate conclusion and providing advice regarding their significance were challenging tasks. These unclassified VUSs are not communicated to the patient or to non-genetic professionals to avoid increasing healthcare utilization and costs associated with unnecessary physician visits and imaging tests [38-41]. Therefore, it is urgent to develop functional studies to evaluate the pathogenicity of a suspected variant and determine its involvement in breast and/or ovarian cancer development. New genome editing techniques enable the assessment of genetic variants, including those of uncertain significance, in a native chromosomal context [42]. For example, in a proof of concept study, known mutations and artificial variants in exon18 *BRCA1* were introduced using the CRISPR/Cas9 technique, resulting in traceable changes in BRCA1 mRNA splicing and abundance [43]. Such an approach, when scaled up, allows the evaluation of the combination effects of variants on splicing and transcript levels in any gene.

The overall mutation prevalence in the combined results of previous targeted genotyping studies [7] and the present amplicon sequencing was >30% in a group of patients with early onset breast/ovarian cancer. However, the negative results of genetic testing may not be universally informative for assessing cancer risk and do not rule out hereditary predisposition. The lack of detection of pathogenic mutations in families affected by an increased risk of breast/ovarian cancer may result not only from misinterpretation of determined variants [44], but also from incorrect selection of index women and/or the genetic analytical range [1]. Additionally, for some populations the large rearrangements in *BRCA1/2* genes have been described in a significant proportion of families with strong breast/ovarian cancer history [45,46]. However, in Poland such testing is not routinely performed since no founder mutations were described [47]. In addition, amplicon sequencing with PGM platform does not seem to be a method ready for searching of *BRCA1/2* large rearrangements in genetic counseling [11,48].

The presence of germline mutations in cancer susceptibility genes other than *BRCA1/2* may also lead to the development of hereditary breast/ovarian cancers. An NGS 25-gene panel revealed that the frequency of mutations in genes other than *BRCA1/2* is 4.3%, of which 3.9% are in genes associated with breast/ovarian cancer [49]. The 4.3% frequency of germline mutations in additional cancer susceptibility genes was also detected through analyses of breast cancers in The Cancer Genome Atlas Project [50]. However, with the exception of *PALB2*, most of these predisposition genes do not reach the same degree of significance as *BRCA1* predisposition [51], and in contrast to *BRCA1/2* mutations, neither age at breast cancer diagnosis nor family history of ovarian or young breast cancer are predictive factors for other mutations [49].

## Conclusions

NGS testing might be routine for many genes, including *BRCA1* and *BRCA2*. However, the cost of complete genetic testing is significantly higher than that of conventional PCR-based genetic testing; the internal costs in our institution, excluding personnel and overhead, are $120 vs. $20 [7]. Therefore, introducing the NGS to routine genotyping of *BRCA* (and other genes) in clinical practice would need additional financial support from public healthcare systems. Instead, targeted screening for specific *BRCA1* mutations performed at the reasonable costs can be offered to all breast or ovarian cancer patients in the Polish population, regardless of the family history or the age of disease onset [7,52-54]. Since PCR-based cost-efficient testing for *BRCA1* and *BRCA2* can detect mainly pre-selected mutations, the more recurrent mutations included in the screening, the greater efficiency of the testing.

In the present study, we identified two mutations, namely, Q563X (*BRCA1*) and N3124I (*BRCA2*), showing a strong founder effect in the Polish patients with a hereditary risk of ovarian/breast cancer. These mutations should be included in the set of mutations analyzed in PCR-based targeted screening programs in Poland. Sanger direct sequencing should be used to confirm the presence of a pathogenic mutation detected by PCR- or NGS-based genotyping, and it is the method of choice for cascade screening of relatives of the index patient. Finally, NGS-based testing of all coding exons in both genes may be reserved for patients with breast or ovarian cancer that is more likely related to *BRCA*. These considerations for the comprehensive assessment of pathogenic mutations in breast and ovarian cancers are exemplified for *BRCA1* C1501X mutation found in multiple family members during conducting this study (Table 5 and Additional file 2). However, further studies are required to determine which clinical features may better define a feasible link between breast/ovarian cancer and *BRCA1*/*2* mutations.

**Table 5:** **Example of multilevel genetic testing workflow that led to identification of** ***BRCA1*** **C1501X mutation in multiple family members during conducting this study**

**Testing round**	**Testing workflow**	**Methods**	**Results**
I	Targeted screening using PCR-based technology	TaqMan SNP Genotyping Assays	Negative for selected variants*
II	Searching for new pathogenic variants using NGS	NGS of *BRCA1*/*BRCA2* exons	Pathogenic variant (C1501X) found
III	Validation and cascade screening	Sanger direct sequencing	Validation in the index patient and mutation found in seven out of eight studied relatives #

*Eleven *BRCA1* mutations (c.66_67delAG, C61R, c.3700_3704del5, c.3756delGTCT, c.3777delT, c.4035delA, c.4041delAG, c.4065delTCAA, c.5263delC, R1738E and R1751X) and nine *BRCA2* mutations (E394X, c.5239insT, c.5946delT, c.5964delAT, c.6447delTA, c.7910del5, c.8924delT, R3128X and c.9402delT) [7,30].

#family tree is depicted in Additional file 2.

## Additional files

Additional file 1:
**Contains Table S1 and** **Table S2 with a list of single nucleotide variants and indels, respectively, found in** ***BRCA1/2*** **genes.**
Additional file 2:
**Family tree harboring C1501X mutation in** ***BRCA1*** **.** BRC; breast cancer, RRM; risk reduction mastectomy, RRA; risk reduction adnexectomy; DLBCL; diffuse large B-cell lymphoma.

## Abbreviations

BRCA1: Breast cancer 1, early onset
BRCA2: Breast cancer 2, early onset
NGS: Next generation sequencing
SNV: Single nucleotide variants
BIC: Breast Information Core
VUS: Variant of uncertain significance
