# Supplementary figures and images for: New recurrent BRCA1/2 mutations in Polish patients with familial breast/ovarian cancer detected by next generation sequencing
Source: BMC Med Genomics. 2015 May 7;8:19. doi: 10.1186/s12920-015-0092-2 (PMC4429836; doi:10.1186/s12920-015-0092-2)

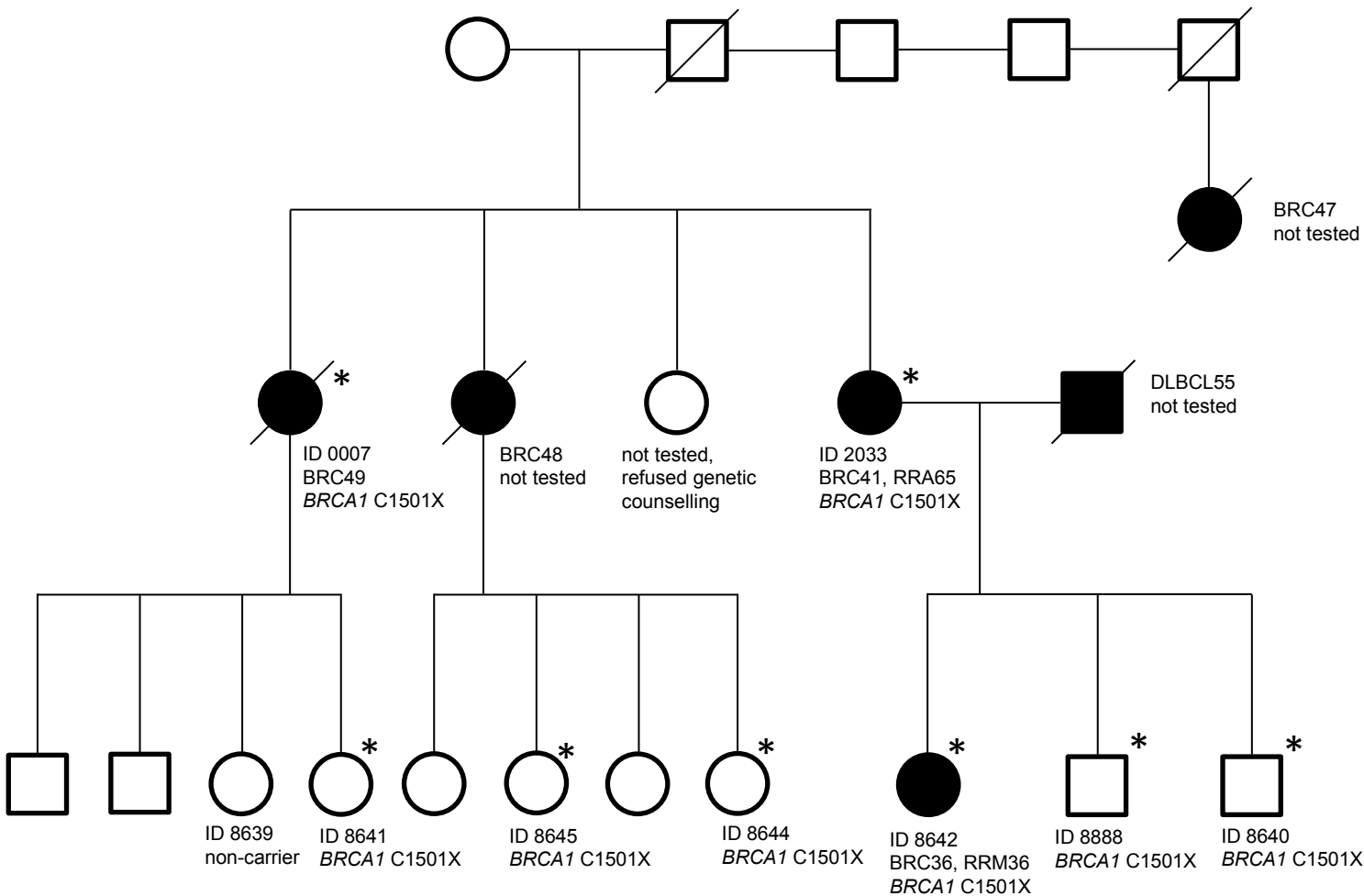

Supplement: Additional file 2: — Family tree harboring C1501X mutation in BRCA1 . BRC; breast cancer, RRM; risk reduction mastectomy, RRA; risk reduction adnexectomy; DLBCL; diffuse large B-cell lymphoma. [file 12920_2015_92_MOESM2_ESM.pdf]
